# Nitrate Nitrogen Addition Promotes Soil Aggregate Stability in *Larix olgensis* Forest

**DOI:** 10.3390/microorganisms14040922

**Published:** 2026-04-19

**Authors:** Tongbao Qu, Yushan Liu, Shilong Xie, Yihao Zhang, Yinglun Sun, Lei Zhao

**Affiliations:** College of Forestry and Grassland, Jilin Agricultural University, Changchun 130118, China; qvtb@jlau.edu.cn (T.Q.); 18943478100@163.com (Y.L.); 18152743686@163.com (S.X.); a2298873304@163.com (Y.Z.); sunyinglun123@163.com (Y.S.)

**Keywords:** nitrogen form, aggregate stability index, microbial community, soil ecological health

## Abstract

Nitrogen addition significantly affects soil aggregate stability by altering the aggregate microenvironment. Although the ecological effects of nitrogen addition on soil aggregates have been extensively investigated, studies specifically focusing on the responses of soil aggregate stability in *Larix olgensis* forest understories remain scarce. The effects of different forms of nitrogen addition (urea (CO(NH_2_)_2_), ammonium chloride (NH_4_Cl), and sodium nitrate (NaNO_3_)) were investigated at 20 kg N·ha^−1^·yr^−1^ across all treatments, and the physicochemical properties, stability, and microbial community composition of soil aggregates were determined to analyze soil aggregate stability. NaNO_3_ significantly increased soil nutrient contents, promoted the formation of macroaggregates, and significantly enhanced soil aggregate stability. NH_4_Cl significantly decreased bacterial diversity in microaggregates, while NaNO_3_ significantly elevated fungal diversity in macroaggregates. CO(NH_2_)_2_ and NH_4_Cl increased the relative abundances of Ascomycota and Proteobacteria in microaggregates, whereas NaNO_3_ elevated the relative abundances of Mortierellomycota and Gemmatimonadetes in soil aggregates of all particle sizes. These results indicated that NaNO_3_ was more effective in improving soil aggregate stability and exerted regulatory effects on microbial community structure compared to the other nitrogen forms. These findings can provide a theoretical basis for an in-depth understanding of the microecological processes of forest soil aggregates under the context of nitrogen deposition.

## 1. Introduction

As the dominant component of terrestrial ecosystems, forest ecosystems serve not only as critical carbon pools in the global carbon cycle but also perform irreplaceable ecological functions, such as sustaining biodiversity and regulating climate [1]. Anthropogenic activities such as fossil fuel combustion and fertilizer application intensify atmospheric nitrogen deposition and profoundly affect the structure and function of forest ecosystems [2,3]. As the central carrier of forest material cycling, soil serves as a critical interface for interactions among plants, microorganisms, and the environment [4]. Soil aggregates, the basic structural units of soil, exhibit physical structure stability that directly determines the long-term sequestration efficiency and turnover processes of key elements including carbon and nitrogen, and serves as the structural foundation for maintaining the functional stability of forest ecosystems [5,6,7]. Most studies focused on the effects of nitrogen addition on carbon and nitrogen storage, enzyme activity and nutrient availability in soil aggregates [8,9,10]; however, systematic knowledge remains lacking regarding how nitrogen inputs with distinct chemical forms specifically influence soil aggregate stability and corresponding mechanisms. Further exploring the effects of different forms of nitrogen addition on the stability of forest soil aggregates and clarifying their internal mechanisms can provide an important theoretical basis for assessing the impacts of atmospheric nitrogen deposition on the stability of forest ecosystems.

The formation and stabilization of soil aggregates constitute a complex process, jointly regulated by biotic and abiotic factors [11]. According to the hierarchical theory of soil aggregate formation, macroaggregates (>0.25 mm) are formed by microaggregates (<0.25 mm) as building blocks, bound together by organic binding agents and fungal hyphae [12]. As the most active biological driving factor, soil microbial communities exert core effects of community characteristics on aggregate stability [13], while aggregates themselves provide microorganisms with microhabitats characterized by diverse physical structures and nutrient conditions [14,15]. Spatially heterogeneous habitats formed by aggregates of different size fractions directly regulate mass exchange, energy flow and community distribution patterns between microorganisms and the environment, and further shape the distribution of microbial communities within aggregates [16,17]. Studies indicated that bacterial biomass and total microbial biomass have been generally more abundant in larger aggregates of 1–2 mm, whereas the relative proportion of fungi tended to increase with decreasing aggregate size [18]. In addition, exogenous nitrogen addition drove the redistribution of microbial communities among different soil aggregate size classes by altering soil physicochemical properties and the particle-size composition of soil aggregates [19,20,21]. For example, AMF communities exhibit taxon-specific responses to ammonium availability and repeated nitrogen inputs, which further contribute to changes in soil aggregation [22]. These findings reveal the microbial spatial patterns shaped by aggregate size differentiation and their response mechanisms to exogenous disturbances. However, the specific manifestations and regulatory pathways of these mechanisms in forest soil aggregates remain unclear.

The *Larix olgensis* is an important fast-growing timber species in Northeast China, characterized by high tolerance to cold climates and excellent wood quality. It is widely planted as a key species for timber production, water conservation, and soil and water retention in the region, and also serves as a critical component of forest ecosystem carbon sinks and nutrient cycling [23]. Existing studies on *L. olgensis* forests confirmed that nitrogen addition can alleviate soil nitrogen limitation by enhancing nitrogen availability, thereby promoting soil nutrient cycling and maintaining ecosystem stability [24]; however, the mechanisms underlying the stability of soil aggregates in *L. olgensis* forests remain unexplored. Elucidating the effects of nitrogen addition of different forms on soil aggregate stability and the associated microbial community composition, and resolving the coupling relationship between the physicochemical properties of soil aggregates and microbial communities, is conducive to an in-depth understanding of the microecological processes of forest soil aggregates under the context of nitrogen deposition, thereby providing a scientific basis for the sustainable management of plantations under global change scenarios.

## 2. Materials and Methods

### 2.1. Experimental Area and Design

This research was conducted in a *L. olgensis* forest, which is located on the campus of Jilin Agricultural University (43°05′–45°15′ N; 124°18′–127°05′ E), Changchun, Jilin Province, China. The terrain of this region is generally higher in the southeast and lower in the northwest, with a relatively flat topography. The soil is classified as loam, consisting of 46.2% sand, 30.4% silt, and 23.4% clay. The initial soil pH in control plots was 5.6. And the elevation ranges from 250 to 350 m. Climatically, it belongs to a mid-latitude temperate monsoon climate, with a mean annual temperature of 4.8 °C and a mean annual precipitation of 570.3 mm; precipitation throughout the year is concentrated in July and August. The understory vegetation is relatively sparse, with an average coverage of only 2–4%, dominated by *Chelidonium majus*, *Viola prionantha*, and *Bromus inermis*.

The nitrogen addition experiment was initiated in 2018 and lasted for seven years until soil sampling in 2025, adopting a completely randomized design. Each plot measured 5 m × 5 m. The perimeter of each plot was demarcated using nylon ropes, and a tag was inserted in the center to distinguish different treatments. A buffer zone 2–5 m wide was set between any two plots to avoid mutual interference. The nitrogen addition experiment comprised three distinct nitrogen application forms: urea (CO(NH_2_)_2_), ammonium chloride (NH_4_Cl), and sodium nitrate (NaNO_3_), representing amide nitrogen, ammonium nitrogen, and nitrate nitrogen, respectively. These three forms cover the main nitrogen components of atmospheric deposition and key available nitrogen types for forest soil ecosystems. The nitrogen application rate was standardized at 20 kg N·ha^−1^·yr^−1^, and the control plots received no nitrogen treatment. The experimental design included one control group and three nitrogen-treated groups, each with three replicates, resulting in a total of 12 plots. Nitrogen addition treatments were conducted twice a year, in May and October. The required fertilizers were thoroughly mixed with the original soil from the plots, after removing stones, dead branches, and fallen leaves, and then uniformly applied to the soil surface.

### 2.2. Soil Sample Collection

Soil sample collection within the quadrats commenced in August 2025. Before sampling, surface litter and weeds were removed from the soil surface. Each experimental plot contained five sampling points. Soil samples were collected from the 0–20 cm soil layer using a 5 cm soil auger at each point; the five samples were then composited into one mixed sample, placed in rigid plastic boxes to prevent compression, stored in dry ice, and immediately transported to the laboratory. After being transported to the laboratory, the soil samples underwent pre-treatment: plant roots and large stones were removed; large soil clods were broken apart along their natural fracture planes to prevent damage to soil aggregates; and the soil was subsequently passed through an 8 mm sieve.

All samples were divided into two subsamples; one subsample was retained as bulk soil, and the remaining subsample was used to separate soil aggregates via dry sieving [25]. First, the soil was air-dried at low temperature until its water content reached 10–15%. Subsequently, an electric sieving machine (Model DM185, Shanghai Dema Information Technology Co., Ltd., Shanghai, China) with sieve sizes of 2 mm and 0.25 mm arranged from top to bottom was used. The soil samples were sieved at a constant frequency of 300 rpm for 5 min to obtain three aggregate size classes: macroaggregates (>2 mm), small aggregates (0.25–2 mm), and microaggregates (<0.25 mm). The weight of each fraction was recorded and used to calculate their relative percentages. All bulk soil and aggregate samples were further divided into two parts. One part was air-dried in a well-ventilated area for the determination of soil organic carbon (SOC), total nitrogen (TN), total phosphorus (TP), soil electrical conductivity (EC), ammonium nitrogen (NH_4_^+^-N), and nitrate nitrogen (NO_3_^−^-N). The other part was stored in a −80 °C refrigerator for the analysis of soil microbial community composition and diversity.

### 2.3. Soil Index Determination Method

#### 2.3.1. Chemical Test Methods of Soil Properties

The EC was determined using a Leici DDS-307A electrical conductivity meter at a soil-to-water ratio of 1:5. NH_4_^+^-N and NO_3_^−^-N contents were measured via a continuous flow analyzer (QC8500, Shanghai, China) [26]. The SOC content was measured by the potassium dichromate-external heating method [27]. The TN content was determined by using j200 laser elemental analyzer [28]. The TP content was measured by molybdenum–antimony colorimetry [29].

#### 2.3.2. Soil Aggregate Stability

Soil aggregate stability is characterized by particle size distribution, geometric mean diameter (GMD), and mean weight diameter (MWD). The calculation formulas are as follows:

(1) $$W_{i}\;=\;m_{i}/M$$

(2) $$GMD=exp\left(\frac{\sum_{i=1}^{n}w_{i}lnx_{i}}{\sum_{i=1}^{n}w_{i}}\right)$$

(3) $$MWD=\sum_{i=1}^{n}X_{i}W_{i}$$

where *W_i_* refers to the mass fraction of aggregates in this size fraction relative to the total mass of aggregates in the soil sample (%), *m_i_* denotes the mass of aggregates in this size fraction, *M* represents the total mass of aggregates in the soil sample, *x_i_* is the mean diameter of aggregates in the *i*-th size fraction (mm), and *n* is the number of aggregate size fractions.

#### 2.3.3. Determination of Soil Microbial Community Structure

Genomic DNA of soil samples was extracted via the CTAB or SDS method. The purity and concentration of the extracted DNA were determined using agarose gel electrophoresis. An appropriate amount of sample DNA was placed in a centrifuge tube and diluted to a concentration of 1 ng/μL with sterile water.

16S rRNA and ITS genes of distinct regions (16SV4/16SV3-V4/16SV4-V5/16SV5-V7, ITS1/ITS2) were amplified using specific primers (e.g., 16SV4: 515F-806R; ITS1: ITS5-1737F-ITS2-2043R) with the barcode. All PCR reactions were carried out with 15 µL of Phusion High-Fidelity PCR Master Mix, 0.2 µM of forward and reverse primers, and about 10 ng template DNA. Thermal cycling consisted of initial denaturation at 98 °C for 1 min, followed by 30 cycles of denaturation at 98 °C for 10 s, annealing at 50 °C for 30 s, and elongation at 72 °C for 30 s and 72 °C for 5 min.

The PCR products were purified using magnetic bead purification. Samples were mixed in equidensity ratios based on the concentration of PCR products. After thorough mixing, the PCR products were detected and target bands were recovered. The sequencing libraries were subjected to indexing and quantification using Qubit and Q-PCR. After the libraries passed quality inspection, sequencing was performed on the Illumina NovaSeq 6000 platform (Illumina, Inc., San Diego, CA, USA). The sequencing work, including the calculation of alpha diversity indices (Chao1, Shannon–Wiener, and Simpson) using QIIME2 2022.2 based on the rarefied OTU table, was commissioned to Novozymes Biotechnology Co., Ltd., Tianjin, China.

### 2.4. Statistical Analysis

Graphs were drawn with Origin 2024. Data statistics and analysis were carried out with SPSS 26.0. Redundancy analysis (RDA) was performed using Canoco 5.0 to explore the relationship between soil environmental factors and microbial community composition. Partial least squares path modeling (PLS-PM) analysis was performed using the R package v4.5.2 ‘plsm’ to evaluate the direct and indirect effects of nitrogen addition in different forms and soil aggregate particle size distribution on soil microbial diversity and community composition.

## 3. Results

### 3.1. Effects of Nitrogen Addition on Soil Aggregate Physicochemical Properties

**Figure 1:**
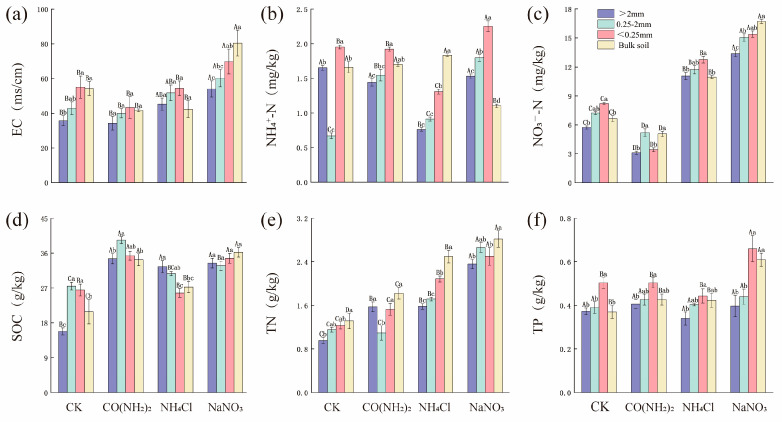
The effects of different forms of nitrogen addition on the physical and chemical properties of soil aggregates. (**a**) Soil EC, (**b**) NH_4_^+^-N, (**c**) NO_3_^−^-N, (**d**) SOC content, (**e**) TN content, (**f**) TP content. Different lowercase letters indicate significant differences among different particle sizes under the same treatment (*p* < 0.05). Different uppercase letters indicate significant differences among different treatments for the same particle size (*p* < 0.05).

Under NaNO_3_ treatment, EC and TP contents showed a clear increasing trend with decreasing aggregate particle size, and were significantly higher than those under other treatments across most aggregate fractions (Table S1; Figure 1a,f). Under NH_4_Cl treatment, NH_4_^+^-N content was observed to be significantly lower in both macroaggregates and microaggregates, but higher in bulk soil. In contrast, NH_4_^+^-N content in microaggregates under the NaNO_3_ was the highest among all treatments (Figure 1b). NO_3_^−^-N contents under NaNO_3_ were significantly higher than those under other treatments across all aggregate fractions, while those under CO(NH_2_)_2_ were consistently lower (Figure 1c). SOC contents in small aggregates under the CO(NH_2_)_2_ were significantly higher than those in other treatments (Figure 1d). Regardless of the form of nitrogen addition, the TN contents in both soil aggregates and bulk soil were significantly increased, with the NaNO_3_ exhibiting the most pronounced effect (Figure 1e). NH_4_Cl significantly decreased the pH value of bulk soil and all particle-size aggregates (Figure S1).

### 3.2. Effects of Nitrogen Addition on Soil Aggregate Stability

In terms of distribution, forestland soil was dominated by small aggregates, while microaggregates accounted for the lowest proportion (Table 1). After the application of nitrogen in different forms, the proportion of small aggregates decreased significantly. Among all nitrogen addition treatments, the NaNO_3_ significantly increased the proportion of macroaggregates, which was 43.21% higher than that in the CK. In terms of soil aggregate stability, the MWD and GMD values of the NaNO_3_ were significantly higher than those in other treatments.

### 3.3. Effects of Nitrogen Addition on Soil Aggregate Microbial Diversity

**Figure 2:**
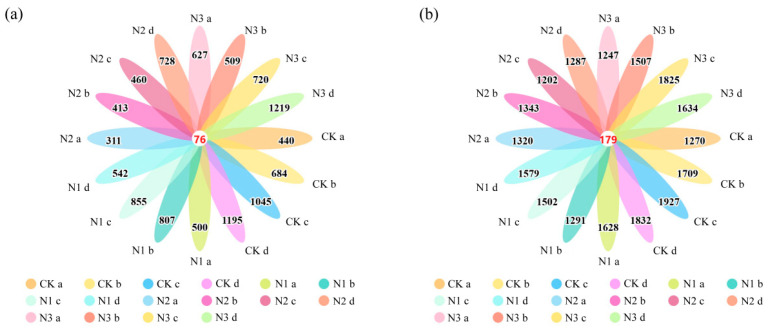
Venn diagram of amplicon sequence variants (ASVs) of fungal and bacterial communities in soil aggregates after addition of different forms of nitrogen. CK: Treatment without nitrogen addition, N1: CO(NH_2_)_2_, N2: NH_4_Cl, N3: NaNO_3_; a: >2 mm, b: 0.25–2 mm, c: <0.25 mm, d: bulk soil; (**a**) fungal community, (**b**) bacterial community.

Fungal ASV richness differed significantly among all groups (Figure 2a). The highest richness was recorded in bulk soil under the NaNO_3_, at 1219, followed by bulk soil under the CK with 1195, while the lowest richness was observed in macroaggregates under the NH_4_Cl, at only 311. Bacterial ASV richness was significantly higher than fungal richness across all treatments (Figure 2b). The highest bacterial values were detected in microaggregates under the CK and NaNO_3_, at 1927 and 1825, respectively; relatively lower values were found in microaggregates under the NH_4_Cl and small aggregates under the CO(NH_2_)_2_, at 1202 and 1291, respectively.

In the absence of nitrogen addition, fungal Chao1, Shannon–Wiener, and Simpson indices increased significantly with decreasing aggregate size, whereas bacterial diversity indices showed no significant differences among aggregate size classes (Table 2 and Table S2). Following exogenous nitrogen input, the CO(NH_2_)_2_ significantly reduced fungal Chao1 indices in macroaggregates and bulk soil, and also significantly decreased Shannon–Wiener indices in bulk soil, but exerted no significant effects on bacterial diversity. The NH_4_Cl showed no significant regulatory effects on fungal diversity, but significantly reduced bacterial Chao1 indices in microaggregates. The NaNO_3_ significantly elevated fungal Chao1 indices in macroaggregates, but exhibited no obvious inhibitory effects on bacterial diversity.

### 3.4. Effects of Nitrogen Addition on Soil Aggregate Microbial Communities

**Figure 3:**
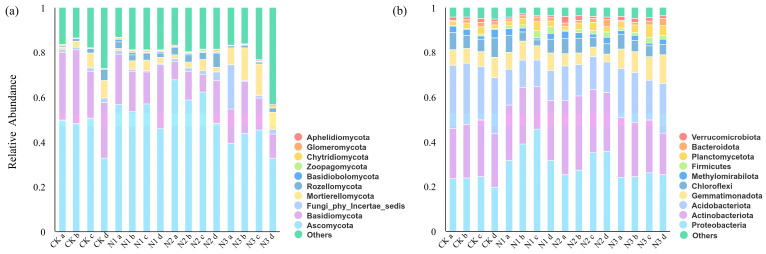
Effects of addition of different forms of nitrogen on microbial community structure in soil aggregates. CK: Treatment without nitrogen addition, N1: CO(NH_2_)_2_, N2: NH_4_Cl, N3: NaNO_3_; a: >2 mm, b: 0.25–2 mm, c: <0.25 mm, d: bulk soil; (**a**) fungal community, (**b**) bacterial community.

At the phylum level, Ascomycota (32.84–67.83%), Basidiomycota (7.81–32.96%), and Mortierellomycota (0.86–14.42%) were the core dominant fungal taxa across all treatments, together accounting for the majority of fungal abundance (Figure 3a). Compared with the CK, Ascomycota abundance was higher under CO(NH_2_)_2_ and NH_4_Cl across all aggregate size classes, with the highest value observed in macroaggregates under NH_4_Cl. In contrast, Ascomycota abundance was lower under NaNO_3_ across all aggregate size classes. Basidiomycota abundance was lower under all nitrogen addition treatments across all aggregate size classes, with the lowest values observed under NH_4_Cl. For Mortierellomycota, abundance was lower under CO(NH_2_)_2_ and NH_4_Cl in bulk soil, while it was higher under NaNO_3_ across all aggregate size classes.

At the phylum level, Proteobacteria (19.91–45.70%), Actinobacteriota (18.51–33.34%), Acidobacteriota (11.93–28.27%), and Gemmatimonadota (3.55–12.70%) were the core dominant bacterial taxa across all treatments, together accounting for the majority of bacterial abundance (Figure 3b). Under CO(NH_2_)_2_, Proteobacteria abundance gradually increased with decreasing aggregate particle size, with the highest value observed in microaggregates. Compared with CK, Proteobacteria abundance was higher under NH_4_Cl in microaggregates and bulk soil, and Actinobacteriota abundance was higher under NH_4_Cl in macroaggregates and small aggregates. In contrast, Gemmatimonadota abundance was higher under NaNO_3_, while no clear differences were observed for Proteobacteria or Actinobacteriota under NaNO_3_ compared to CK. Acidobacteriota abundance was lower under all nitrogen addition treatments across all aggregate size classes.

### 3.5. Relationship Between Soil Physicochemical Properties and Soil Microorganisms in Aggregates of Different Sizes

**Figure 4:**
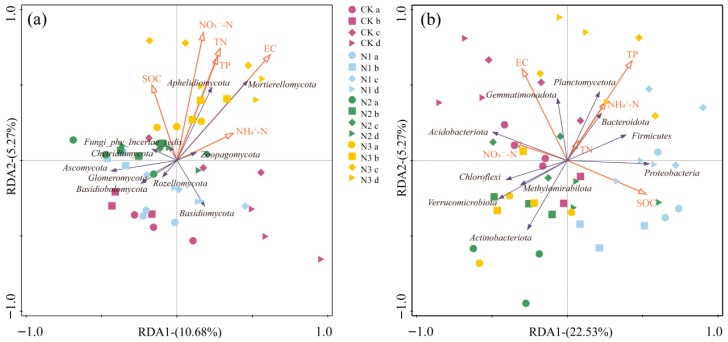
(**a**) Redundancy analysis (RDA) of dominant fungal taxa and soil environmental factors after nitrogen addition. (**b**) RDA of dominant bacterial taxa and soil environmental factors after nitrogen addition. CK: Treatment without nitrogen addition, N1: CO(NH_2_)_2_, N2: NH_4_Cl, N3: NaNO_3_; a: >2 mm, b: 0.25–2 mm, c: <0.25 mm, d: bulk soil.

Redundancy analysis (RDA) was performed separately on soil fungal (Figure 4a) and bacterial (Figure 4b) communities. Results showed that RDA1 and RDA2 together explained 15.59% of the total variation in fungal communities, whereas RDA1 and RDA2 jointly explained 27.80% of the variation in bacterial communities, indicating that environmental factors exerted a significantly stronger driving effect on bacterial community differentiation than on fungal community differentiation. Among the environmental factors, NO_3_^−^-N and EC showed the strongest correlations with fungal community structure, while TP, EC, and SOC were the dominant factors correlated with bacterial community structure. In both fungal and bacterial communities, samples from the NaNO_3_ treatment were distributed mostly along the positive direction of RDA1 and overlapped highly with TN, TP and EC, indicating the highest levels of soil nutrients and EC under this treatment. Under the same treatment, microaggregates were positioned closest to the positive direction of RDA1, and macroaggregates closest to the negative direction, suggesting that microaggregates were most sensitive to changes in soil chemical properties induced by fertilization, whereas macroaggregates exhibited a stronger environmental buffering effect; this pattern was more pronounced in bacterial communities. Within fungi, Mortierellomycota and Aphelidiomycota showed strong positive correlations with NO_3_^−^-N, TN, TP and EC, and were relatively enriched in microaggregates and bulk soil under the NaNO_3_ treatment. Within bacteria, Planctomycetota, Bacteroidota and Firmicutes exhibited strong positive correlations with TP, NH_4_^+^-N and TN, while taxa including Acidobacteriota and Chloroflexi were enriched in macroaggregates under the NH_4_Cl treatment.

### 3.6. Partial Least Squares Path Modeling (PLS-PM) Analysis

**Figure 5:**
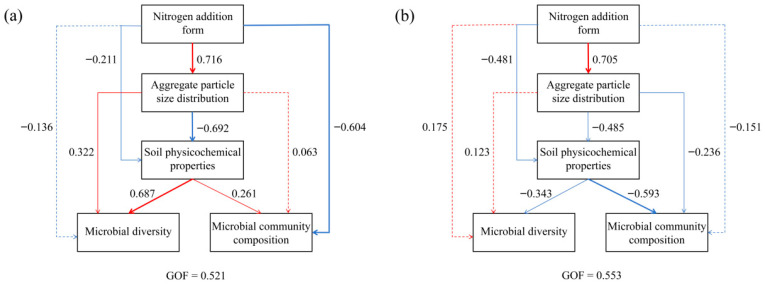
Partial least squares path modeling (PLS-PM) diagram of the effects of different forms of nitrogen addition on fungal (**a**) and bacterial (**b**) diversity and community structure. Red and blue arrows indicate positive and negative correlations, respectively, while dashed arrows indicate no significant correlation (*p* < 0.05).

Partial least squares path modeling (PLS-PM) analysis indicated that the goodness of fit (GOF) of the fungal model was 0.521 (Figure 5a). Nitrogen addition form exerted a significant positive effect on aggregate size distribution, with a path coefficient of 0.716, and indirectly drove changes in soil aggregate physicochemical properties, which in turn positively regulated fungal diversity and community composition. Meanwhile, nitrogen addition form also directly imposed a significant negative effect on fungal community composition, with a path coefficient of −0.604. The GOF of the bacterial model was 0.553 (Figure 5b). Nitrogen addition form likewise imposed a significant positive effect on aggregate size distribution, with a path coefficient of 0.705, whereas aggregate size distribution exhibited a significant negative effect on soil physicochemical properties, with a path coefficient of −0.485. Further analysis demonstrated that soil physicochemical properties ultimately exerted significant negative regulatory effects on bacterial diversity and community composition, with path coefficients of −0.343 and −0.593, respectively. Nitrogen addition form showed no direct effects on bacterial communities.

## 4. Discussion

### 4.1. Changes in the Physicochemical Properties of Soil Aggregates Following Nitrogen Addition

The experiment indicated that the increasing trend of EC and TP contents with decreasing aggregate particle size was particularly pronounced under NaNO_3_. This is most likely attributed to microaggregates possessing a larger specific surface area and stronger adsorption capacity [30,31]. Microaggregates are generally rich in clay minerals and metal oxides, which can immobilize more soluble ions and phosphates through electrostatic adsorption or ligand exchange [32]. Nitrogen addition may further exacerbate nutrient enrichment in microaggregates by altering soil solution ionic strength or influencing microbe-mediated phosphorus activation processes [33]. Moreover, the application of NH_4_Cl significantly decreased the pH value of both bulk soil and all particle-size aggregates. This acidifying effect is immediate, resulting from the release of H^+^ during microbial nitrification of NH_4_^+^, and may intensify over the long term with repeated NH_4_^+^-based fertilizer applications, potentially leading to base cation leaching and soil acidification [34].

This study found that NH_4_Cl significantly decreased NH_4_^+^-N content in macroaggregates and microaggregates, whereas NaNO_3_ significantly increased NH_4_^+^-N content in microaggregates, and NO_3_^−^-N content was significantly reduced under CO(NH_2_)_2_. These results suggest that different nitrogen sources may influence the microdomain distribution of soil nitrogen within aggregates. The observed patterns could be related to the combined effects of aggregate structural stability, cation exchange, and microbial nitrogen transformation processes, but further experimental validation is needed. NH_4_^+^-N can be rapidly immobilized by soil clay minerals and organic matter via cation exchange, thereby accumulating extensively in the soil nitrogen pool [35]. In microaggregates, the smaller pore size and reduced aeration may create localized anaerobic conditions that favor denitrification, leading to lower NO_3_^−^-N accumulation and relatively higher NH_4_^+^-N retention compared to macroaggregates [36]. CO(NH_2_)_2_ must be transformed through the relatively slow mineralization–nitrification process, whose rate is constrained by environmental conditions and microbial activity [37].

The results showed that nitrogen addition significantly increased TN content in both aggregates and bulk soil, with the most pronounced effect observed under NaNO_3_, which is consistent with most previous studies [38]. Due to its high mobility, nitrate-N can diffuse more rapidly among different particle-size fractions in soil and be adsorbed by clay minerals or organic matter, thereby leading to a stronger short-term total nitrogen accumulation effect [39]. The SOC content in small aggregates under CO(NH_2_)_2_ was significantly higher than that in other treatments. This is likely attributable to the combined effects of urea-induced stimulation of microbial assimilation, leading to enhanced microbial necromass production. However, the physical protection mechanisms of small aggregates, such as adsorption onto clay minerals and occlusion within microaggregates, may have played an equally important role in stabilizing the accumulated organic matter [40].

### 4.2. Changes in the Stability of Soil Aggregates Following Nitrogen Addition

MWD and GMD, calculated based on aggregate mass percentage, are important indicators for assessing soil aggregate stability [30]. Improved soil aggregate stability typically indicates stronger soil erosion resistance and better physical protection capacity of soil organic carbon [41]. Our experimental results showed that NaNO_3_ significantly increased the proportion of soil macroaggregates, as well as MWD and GMD, which is consistent with the study by Riggs et al. [42]. This may be attributed to NaNO_3_, as an easily available nitrogen source, more effectively stimulating fungal activity in soil microorganisms; this promotes mycelial entanglement and the secretion of microbial metabolic products, thereby enhancing the cohesion of soil particles [43]. In contrast, the promoting effects of CO(NH_2_)_2_ and NH_4_Cl on macroaggregates were not significant, which may be related to their different transformation pathways and differential impacts on microbial community structure. CO(NH_2_)_2_ requires mineralization, whereas ammonium nitrogen may generate acidity through nitrification, potentially adversely affecting certain aggregate binding materials [44]. Additionally, nitrogen addition consistently reduced the proportion of small aggregates. This pattern could be partially explained by the fact that exogenous nitrogen inputs may alter organic matter turnover and soil physicochemical conditions, thereby influencing the redistribution and reorganization of soil aggregates across different size classes [12].

### 4.3. Changes in Soil Aggregate Microbial Diversity Following Nitrogen Addition

Soil microorganisms, as the most active core components in the soil ecosystem, are not only the core drivers of soil nutrient cycling and carbon cycling but also regulate various biological and abiotic processes in soil, thus playing an irreplaceable and crucial role in the global carbon and nitrogen balance [45]. The results showed that in the absence of exogenous nitrogen addition, fungal α-diversity indices significantly increased with the decrease in aggregate particle size; in contrast, there was no significant difference in bacterial α-diversity among aggregates of different particle sizes. This finding is consistent with previous studies that fungal communities often exhibit stronger spatial heterogeneity and habitat preference [46]. Microaggregates provide more diverse physical microenvironments and organic matter compositions, offering favorable habitats for the formation of fungal hyphal networks and the maintenance of their functions, thereby supporting higher fungal species richness and evenness [47]. Bacterial communities are more uniformly distributed among different aggregate habitats and their sensitivity to microenvironmental heterogeneity is lower than that of fungi [48].

Our studies demonstrated that exogenous nitrogen addition, such as urea and ammonium chloride, led to significant decreases in fungal Chao1 and Shannon indices in macroaggregates and bulk soil under CO(NH_2_)_2_ treatment, whereas NaNO_3_ significantly increased the fungal Chao1 index in macroaggregates. This may be attributed to the ammonia released during urea hydrolysis causing a localized pH spike in the soil, disrupting fungal cell wall stability and inhibiting spore germination and mycelial growth, and the high aeration in macroaggregates further intensifies the inhibitory effect on fungal diversity [49]. The readily available NO_3_^−^ provided by NaNO_3_ satisfies the nitrogen requirements of heterotrophic fungi within large aggregates, and combined with the abundant readily decomposable organic matter in these aggregates, this synergistically promotes fungal colonization and proliferation, thereby enhancing species richness [50]. In addition, the sodium ion (Na^+^) introduced by NaNO_3_ application may also affect soil aggregation. Sodium ions can influence soil particle flocculation and dispersion, thereby affecting the combination and stability of soil aggregates, which in turn indirectly affects fungal habitat conditions and diversity [51]. In contrast to the response of fungi, CO(NH_2_)_2_ and NaNO_3_ had no significant effects on bacterial diversity, whereas NH_4_Cl significantly reduced the bacterial Chao1 index in microaggregates. Ammonia released from CO(NH_2_)_2_ hydrolysis can be utilized by some bacteria, and NO_3_^−^ supplied by NaNO_3_ can also meet their nitrogen demand [52]. Therefore, neither of these two treatments exerted obvious regulatory effects on bacterial diversity. The inhibitory effect of NH_4_Cl on bacterial diversity in microaggregates was mainly attributed to the small pore size and poor permeability of microaggregates, which led to the accumulation of Cl^−^ and aggravated osmotic pressure and acid stress.

### 4.4. Changes in Soil Aggregate Microbial Community Composition Following Nitrogen Addition

Our results showed that compared with the control group (CK), CO(NH_2_)_2_ increased the relative abundance of Ascomycota in aggregates of all size fractions, and NH_4_Cl elevated the abundance of this phylum in macroaggregates. This may be related to the preference or tolerance of certain Ascomycota taxa for ammonium nitrogen. The ammonium nitrogen derived from urea hydrolysis and that directly supplied by ammonium chloride both provided sufficient nitrogen sources for its growth and reproduction [53]. In this experiment, NaNO_3_ decreased the relative abundance of Ascomycota in aggregates of all size fractions. This may be related to the high mobility, poor retention and high energy consumption for transformation of nitrate nitrogen [51]. This experiment demonstrated that all nitrogen addition treatments reduced the relative abundance of Basidiomycota in aggregates of all size fractions, with NH_4_Cl exhibiting the most pronounced inhibitory effect. This may be because soil acidification induced by NH_4_Cl further disrupted the neutral microenvironment preferred by Basidiomycota and ultimately inhibited their growth and reproduction [54]. The relative abundance of Mortierellomycota in aggregates of all size fractions was increased under NaNO_3_. Nitrate nitrogen may stimulate the enhanced organic matter decomposition activity of this microbial group for acquiring carbon skeletons by altering soil C:N stoichiometry. This allows them to gain a competitive advantage in microbial interactions [55].

Our results showed that under CO(NH_2_)_2_, the relative abundance of Proteobacteria increased with the decrease in aggregate particle size, and increase in microaggregates. This may be related to the strong adsorption capacity of microaggregates for ammonium nitrogen, as well as the relatively high content of labile carbon and nitrogen substrates often associated with microaggregates [56]. NH_4_Cl also increased the abundance of Proteobacteria in microaggregates and bulk soil, further confirming the preference of these microorganisms for ammonium nitrogen. The reason is that Proteobacteria include a large number of fast-growing eutrophic bacteria that can quickly respond to directly assimilable ammonium nitrogen, and the relatively abundant organic matter and good physical protection in microaggregates together provide a suitable microhabitat for their proliferation [57,58]. Actinobacteriota was enriched in macroaggregates and microaggregates under NH_4_Cl. This may be attributed to their tolerance to acidic environments and metabolic capacity to utilize complex organic matter. The ammonium nitrogen provided by NH_4_Cl may also stimulate their degrading enzyme activity. Meanwhile, the acidification stress weakens some competing microbial groups, allowing Actinobacteriota to gain an advantage in this ecological niche [59]. Experimental results indicated that the relative abundance of Gemmatimonadota was enriched under NaNO_3_. Gemmatimonadota is generally considered an oligotrophic (K-strategist) bacterial group, adept at surviving in environments with intense resource competition [60]. Nitrate nitrogen has stable chemical properties and does not easily cause severe acidification; this environment may be more favorable for microbial groups with specific metabolic strategies such as Gemmatimonadota [51]. They may efficiently utilize nitrate nitrogen and couple it with the decomposition of specific carbon sources, thereby gaining an advantage in the community. Nitrogen treatment uniformly reduced the abundance of Acidobacteria in aggregates across all particle size fractions. This may be attributed to the widespread elevation of soil available nitrogen levels, which imposes broad negative selection pressure on oligotrophic bacteria adapted to low-nutrient environments, thereby driving the overall bacterial community toward a eutrophication strategy [61].

## 5. Conclusions

This study systematically investigated the effects of nitrogen addition in different forms on the physicochemical properties, aggregate characteristics, and microbial community structure of forest soil aggregates. The results showed that nitrate nitrogen significantly increased soil EC, TP, and nitrogen contents, while also effectively promoting the formation of macroaggregates and enhancing aggregate stability. Microbial communities exhibited taxon-specific responses to nitrogen forms: sodium nitrate improved fungal diversity in macroaggregates as well as the abundances of Mortierellomycota and Gemmatimonadota without exerting inhibitory effects on bacteria; urea reduced fungal diversity in macroaggregates and bulk soil; ammonium chloride inhibited bacterial diversity in microaggregates and the abundance of Basidiomycota; all nitrogen addition treatments suppressed the abundance of Acidobacteriota. Overall, NaNO_3_ showed more pronounced positive effects on soil aggregate stability indicators, soil nitrogen availability, and microbial community composition. These findings provide observational support for understanding the relationships among nitrogen deposition, soil aggregates, and microorganisms in forest ecosystems, and offer a reference for the management of forest soil nutrients and the sustainable development of planted forests.

## Figures and Tables

**Table 1:** Effects of different forms of nitrogen addition on soil aggregate stability.

Treatment	Soil Aggregate Size (%)			MWD (mm)	GMD (mm)
	>2 mm	0.25–2 mm	<0.25 mm		
CK	33.23 ± 4.64 b	61.85 ± 5.39 a	3.74 ± 0.99 a	1.70 ± 0.08 b	1.43 ± 0.03 b
CO(NH_2_)_2_	35.81 ± 0.59 b	59.83 ± 1.53 a	4.08 ± 0.94 a	1.75 ± 0.01 b	1.46 ± 0.02 b
NH_4_Cl	36.48 ± 5.01 b	58.82 ± 5.53 a	4.14 ± 0.28 a	1.76 ± 0.09 b	1.47 ± 0.08 b
NaNO_3_	47.59 ± 7.99 a	48.42 ± 8.66 b	3.07 ± 1.85 a	1.98 ± 0.14 a	1.68 ± 0.16 a

Note: Different lowercase letters indicate significant differences among different treatments for the same particle size (*p* < 0.05).

**Table 2:** Effects of different forms of nitrogen addition on microbial diversity in soil aggregates.

		Treatment	Soil Aggregate Size			Bulk Soil
			>2 mm	0.25–2 mm	<0.25 mm	
Fungal	Chao1 Index	CK	792 ± 172 ab	1065 ± 187 a	1582 ± 315 a	1568 ± 135 a
		CO(NH_2_)_2_	662 ± 274 b	1011 ± 486 a	1104 ± 546 a	893 ± 316 b
		NH_4_Cl	735 ± 35 ab	880 ± 270 a	930 ± 568 a	1194 ± 386 ab
		NaNO_3_	1111 ± 221 a	1083 ± 371 a	1214 ± 402 a	1558 ± 210 a
	Shannon–Wiener Index	CK	4.57 ± 1.97 a	5.23 ± 1.29 a	7.02 ± 0.49 a	6.6 ± 1.18 a
		CO(NH_2_)_2_	5.11 ± 1.43 a	6.34 ± 0.63 a	6.61 ± 1.24 a	4.61 ± 0.79 b
		NH_4_Cl	4.79 ± 1.16 a	5.84 ± 0.36 a	6.48 ± 0.65 a	6.2 ± 0.75 a
		NaNO_3_	5.17 ± 1.62 a	6.01 ± 0.7 a	6.64 ± 0.32 a	7.49 ± 0.14 a
	Simpson Index	CK	0.76 ± 0.25 a	0.83 ± 0.14 a	0.96 ± 0.02 a	0.91 ± 0.1 ab
		CO(NH_2_)_2_	0.9 ± 0.05 a	0.94 ± 0.01 a	0.96 ± 0.03 a	0.85 ± 0.05 b
		NH_4_Cl	0.83 ± 0.09 a	0.91 ± 0.06 a	0.96 ± 0.01 a	0.95 ± 0.02 ab
		NaNO_3_	0.85 ± 0.17 a	0.93 ± 0.03 a	0.96 ± 0.01 a	0.98 ± 0 a
Bacterial	Chao1 Index	CK	1701 ± 246 a	1891 ± 449 a	1739 ± 118 ab	1772 ± 446 a
		CO(NH_2_)_2_	2077 ± 554 a	1557 ± 335 a	1373 ± 309 ab	1538 ± 198 a
		NH_4_Cl	1578 ± 39 a	1468 ± 337 a	1318 ± 331 b	1222 ± 379 a
		NaNO_3_	1539 ± 211 a	1782 ± 291 a	1818 ± 156 a	1524 ± 131 a
	Shannon–Wiener Index	CK	9.21 ± 0.15 a	9.53 ± 0.34 a	9.62 ± 0.13 a	9.45 ± 0.55 a
		CO(NH_2_)_2_	9.08 ± 0.87 a	8.51 ± 0.99 a	7.91 ± 1.65 a	8.73 ± 0.81 a
		NH_4_Cl	8.97 ± 0.55 a	8.82 ± 1.07 a	8.56 ± 1.22 a	7.99 ± 1.59 a
		NaNO_3_	9.02 ± 0.43 a	9.53 ± 0.38 a	9.61 ± 0.43 a	9.18 ± 0.47 a
	Simpson Index	CK	1 ± 0 a	1 ± 0 a	1 ± 0 a	1 ± 0 a
		CO(NH_2_)_2_	0.98 ± 0.02 a	0.97 ± 0.03 a	0.94 ± 0.06 a	0.98 ± 0.03 a
		NH_4_Cl	0.99 ± 0.01 a	0.99 ± 0.01 a	0.99 ± 0.02 a	0.97 ± 0.04 a
		NaNO_3_	0.99 ± 0.01 a	1 ± 0 a	1 ± 0 a	1 ± 0 a

Note: Different lowercase letters indicate significant differences among different treatments for the same particle size (*p* < 0.05).

## Data Availability

The original contributions presented in this study are included in the article/Supplementary Materials. Further inquiries can be directed to the corresponding author.

## Supplementary Materials

The following supporting information can be downloaded at: https://www.mdpi.com/article/10.3390/microorganisms14040922/s1, Table S1: Two-way ANOVA on the physicochemical properties of soil aggregates. Table S2: Two-Way ANOVA on the Microbial Diversity of Soil Aggregates; Figure S1: Effects of different nitrogen forms on pH value of soil aggregates.
