# Supplementary material for: Nitrate Nitrogen Addition Promotes Soil Aggregate Stability in Larix olgensis Forest
Source: Microorganisms. 2026 Apr 19;14(4):922. doi: 10.3390/microorganisms14040922 (PMC13118409; doi:10.3390/microorganisms14040922)
Supplement: Supplementary file 1 [file microorganisms-14-00922-s001.zip › microorganisms-4169475-supplementary.pdf]

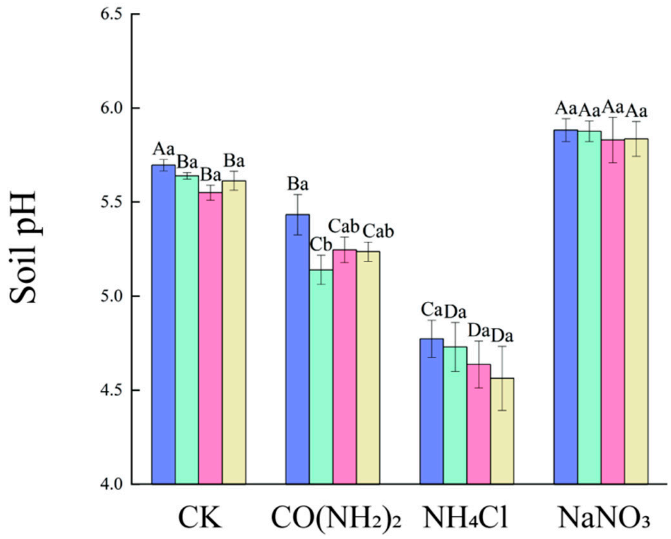

Figure S1. Effects of different nitrogen forms on pH value of soil aggregates.

Table S1. Two-Way ANOVA on the Physicochemical Properties of Soil Aggregates

Factors	Nitrogen Addition form		Soil aggregate size		Nitrogen Addition form × Soil aggregate size	
	F	*P*	F	*P*	F	*P*
EC	65.493	0.000**	19.9	0.000**	5.043	0.000**
NH_4^+-N	86.636	0.000**	142.662	0.000**	79.416	0.000**
NO_3^--N	1124.792	0.000**	28.515	0.000**	9.705	0.000**
SOC	210.885	0.000**	14.020	0.000**	18.525	0.000**
TN	445.913	0.000**	59.913	0.000**	11.696	0.000**
TP	39.519	0.000**	49.299	0.000**	8.045	0.000**

* Indicates significant correlation at the 0.05 level, ** indicates high correlation at the 0.01 level.

Table S2. Two-Way ANOVA on the Microbial Diversity of Soil Aggregates

	Factors	Nitrogen Addition form		Soil aggregate size		Nitrogen Addition form × Soil aggregate size	
		F	*P*	F	*P*	F	*P*
Fungal	Chao1	3.601	0.024*	4.748	0.008**	0.801	0.618
	Shannon	0.904	0.450	6.273	0.002**	1.352	0.251
	Simpson	0.950	0.428	3.725	0.021*	0.679	0.722
Bacterial	Chao1	3.123	0.039*	1.155	0.342	1.136	0.367
	Shannon	3.706	0.021*	0.242	0.866	0.652	0.745
	Simpson	4.012	0.016*	0.459	0.712	0.782	0.634

* Indicates significant correlation at the 0.05 level, ** indicates high correlation at the 0.01 level.
